# Leprosy in the Baltic Provinces

**Published:** 1899-09-23

**Authors:** 


### LEPROSY IN THE BALTIC PROVINCES.

From a Correspondent.

Public attention has been called to the presence of leprosy in the Baltic provinces by a semi-official report recently published in the "St. Petersburg Herold." It appears that in 1867 the first cases of leprosy were discovered in the province of Livonia, in the neighbourhood of Jurjeff, formerly known as Dorpat. In 1870 Dr. Bergmann discovered a little band of lepers near Lake Peipus, in the district of Dorpat, and in 1898 there were 743 lepers in the Government of Livonia. On the initiative of Professor Wahl steps had been taken, meanwhile, to investigate the causes of this horrible scourge, and the result was that an Anti-Leprosy Association was founded, which undertook to isolate the sufferers, and to tend them in leper hospitals. The statutes of this society were confirmed on April 4th, 1891, and since that date a properly organised struggle has been maintained with leprosy in the Baltic Governments. From 1891 to 1896 leper hospitals were founded in Riga, Jurjeff, Nennal, and Wenden, of which the Jurjeff hospital contains 18 beds, and the others from 60 to 80. In 1897 an Imperial grant of 25,000 roubles was made to the society, which was thus enabled to build a fifth hospital in the parish of Tarvasr, in the Felliner district. The total number of beds in the five hospitals now amounted to 308. In 1896 the Livonia Landtag came to the assistance of the society by passing a resolution that the expenses entailed by treating the lepers from the various parishes should be borne by the public funds. It will be seen that a great deal has been done for the lepers in Livonia, and that the precautionary measures thus taken are sufficient for ensuring proper care and treatment for all lepers.

Unfortunately, there is a far less favourable state of matters on the Island of Oesel, lying at the entrance of the Gulf of Riga, which belongs to Livonia. This island has not the necessary means for building a leper hospital. In 1889 the Landrath of Oesel petitioned the Imperial Government for a grant of money and building material from the Crown forests for the purpose of building the hospital required. The necessary timber was granted, but the Medical Department refused to grant the sum of 7,719 roubles asked for. Thus, even the further steps taken by the leading people in Oesel have remained until to-day without any practical result. However, there are now hopes that the leprosy question in Oesel will soon be settled in a satisfactory manner. During the last summer the Society for Maintaining the Public Health sent Dr. A. A. Kobylin to Oesel in order to ascertain to what extent leprosy prevailed there, and what means were necessary for successfully contending with it. It may be expected that Dr. Kobylin's mission will be followed by good results. Leprosy spreads quickly in Oesel. From 1890 to 1896 the number of lepers varied from 22 to 25; but on January 13th, 1898, this number had risen to 96. In a third of the cases the disease is of such a nature as to suggest the probability of its arising from contagion, and thus it seems necessary that the lepers should be isolated. So long as this is not the case there is the continual danger of the sufferers spreading the disease in other Governments, as many Oesel peasants leave the island in summer, and go to the Government of Esthonia and to St. Petersburg in search of work.

A peculiarity of leprosy in the Baltic Provinces is that it makes its appearance in the most unlooked for places. A striking case is reported as having occurred at St. Petersburg during the height of the last winter season. At a ball in that city a dancer suddenly ceased dancing and requested his partner to lead him at once to her chaperon, who turned out to be her father, a Russian official of high standing in the Baltic Provinces. The Russian gentleman then made the unusual request that the young lady should be taken home immediately, and he promised to call on the morrow and make the necessary explanation. Next day he called on the officer and was accompanied by the leading leprosy specialist in St. Petersburg. His explanation was to the effect that he was a physician and while dancing with the young lady he had observed that she was afflicted with leprosy, and that he had therefore taken the liberty of bringing a specialist to refute or confirm his suspicions. To the father's dismay these suspicions proved only too true, and the young lady was at once sent to a leper hospital.

